# Identifying migrant remains in South Texas: policy and practice

**DOI:** 10.1080/20961790.2018.1497437

**Published:** 2018-10-29

**Authors:** M. Katherine Spradley, Nicholas P. Herrmann, Courtney B. Siegert, Chloe P. McDaneld

**Affiliations:** Department of Anthropology, Texas State University, San Marcos (TX), USA

**Keywords:** Forensic anthropology, migrant, migration, positive identification, geophysical survey

## Abstract

In 2012, Texas surpassed Arizona in migrant deaths. The majority of deaths occurred in the Rio Grande Valley, specifically in Brooks County, Texas. Brooks County is one of the poorest in the state and was overwhelmed with deaths, without appropriate resources to follow the state laws pertaining to the investigation of unidentified human remains. Until 2013, most remains that were not immediately identified were buried without collecting DNA samples and the location of burials was not recorded. Our paper outlines the difficulties searching for these burials, the struggles of the families of the missing, and the collaborative approaches to facilitating identifications in South Texas. Community outreach combined with geophysical surveys guide which cemeteries are in need of exhumations. Once cemeteries are surveyed, archaeological methods are employed to exhume remains and document burials. Remains are taken to the Forensic Anthropology Center at Texas State for processing, analysis, and identification efforts. Undergraduate and graduate students clean remains and wash clothing and personal effects.

After skeletal analysis, all information regarding the remains, including photographs of personal effects, are uploaded to the National Missing and Unidentified Persons System (NamUs) and a DNA sample is submitted to the University of North Texas for inclusion in the Combined DNA Index System (CODIS) DNA database. However, CODIS lacks DNA family reference samples from many families of the missing due to families living outside the US or because they do not feel comfortable providing a DNA sample in the presence of law enforcement. Therefore, it is necessary to work with non-governmental organizations who specialize in collecting missing persons reports and DNA samples from the families of the missing. Working collaboratively with multiple agencies, identification of migrant remains is possible.

## Introduction

In 1994, President Bill Clinton enacted a United States Border Patrol (USBP) strategy called “Prevention through Deterrence” in an attempt to deter migration at the US/Mexico border. This strategy funnelled migrants into Arizona’s Sonora Desert resulting in thousands of migrant deaths in the years that followed. Between 1991 and 2012, a total of 2238 migrants died attempting to cross the border in Arizona and is likely an underestimation of actual deaths [1]. Through statistics at the Pima County Office of the Medical Examiner, the majority of the migrants who died in Arizona are mostly male, from Mexico, and between the ages of 18 and 45, fitting the demographics of labourers [2].

In 2012, Texas surpassed Arizona in migrant deaths for the first time with a surge of migrants fleeing violence from Central America. Further, according to USBP statistics, Texas has a higher percentage of females and unaccompanied minors that attempt to cross the border [3]. For the migrants that died in Texas, procedures surrounding the medico-legal investigation are quite different from Arizona. In Arizona, there are no state laws pertaining to unidentified human remains [4]. Meanwhile in Texas, there are state laws that pertain specifically to unidentified human remains. The Texas Criminal Code of Procedures (TCCP) Chapters 49 and 63 states that unidentified human remains shall have an inquest and DNA samples shall be taken and submitted to a state lab for inclusion into a federal DNA database, although the laws are not systematically followed by many counties. In Arizona, 95% of deaths found in close proximity to the border are seen by a medical examiner [4]. It is unknown how many deaths are seen by a medical examiner or forensic pathologist in Texas as this information is not tracked using a centralized system. Arizona is 91% public land, making searching for human remains in the desert near the border more accessible. Texas is 96% private land; therefore, landowner permission is needed to search for human remains and for various reasons permission is not always granted.

The purpose of this article is to focus on identification strategies for migrants that die in South Texas. Because not all unidentified human remains are sent to a medical examiner’s office, positive identification and repatriation is challenging.

## Brooks County, Texas: the epicentre of the mass disaster

On May 1, 2013, a woman named Marta Iraheta, spoke to a crowd in Houston about her nephew Elmer who was dehydrated, hurt, and left to die in the South Texas desert [5]. Elmer was found on Palo Blanco ranch in July of 2012 and buried in the Brooks County cemetery, Sacred Heart Burial Park, in Falfurrias, Texas, with a temporary grave marker. Marta called the Colibrí Center for Human Rights to see if they could help, at which time she informed Colibrí that Elmer was buried at Sacred Heart and the funeral home was going to charge her thousands of dollars for exhumation, several hundred dollars to take a DNA sample, and $100.00 per day to store the remains until Elmer could be repatriated to El Salvador [6]. While plans were made by several local universities to exhume Elmer’s remains and a non-governmental organization agreed to pay for DNA profiling and genetic comparison so that Elmer could be positively identified, no one could remember where Elmer was buried.

In Brooks County, remains of suspected migrants were sent to a funeral home for autopsy services and identification efforts from 2006 to 2013. If the remains were not identified by the funeral home, they were buried. The Justices of the Peace in Brooks County have legal jurisdiction over the unidentified human remains and by state law are required to keep track of the final disposition of the remains. Despite being the law, recording the final disposition of unidentified remains was not enforced, and no one tracked where Elmer or any other human remains were buried. In 2012, grassroots organizations, including the Migrant Right’s Collective, began protesting in Brooks County and were specifically concerned that unidentified human remains were not being sampled for DNA. Eventually, protests and lobbying of state senators by these non-governmental organizations allowed Brooks County to obtain additional funding to send all unidentified human remains to the Webb County Medical Examiner’s Office, located approximately an hour and a half away. Brooks County is among the poorest counties in Texas and did not have the funds to pay for transportation and contracted autopsy services prior to funding from the state. Although unidentified remains have routinely been seen by a medical examiner since 2013, the practice of burial at Sacred Heart without DNA sampling and without recording the specific location of burials since 2006 (potentially earlier, but records are not available) has left many long-term dead buried somewhere in the cemetery without the possibility of identification and repatriation.

Elmer disappeared in 2012, the year that Brooks County recorded approximately 130 deaths. From 2009 (the year that Sheriff’s Office began to collect searchable records) through 2013, the county found and buried 326 individuals. It is unknown how many individuals were buried in the years prior to official record keeping. While the TCCP mandates that the final disposition of unidentified human remains must be recorded by the Justice of the Peace and documented on a death certificate, Elmer’s burial along with all the other unidentified burials were not recorded. Although temporary markers were used to mark some graves, the riding lawn mowers used to maintain the cemetery often displace the markers and they may be replaced in the wrong location or never replaced at all. Even though it was known that Elmer was buried in Sacred Heart, it was not possible to locate his grave due to lack of burial records for the cemetery and burial practices within the county.

What happened to Elmer is representative of what happened to most of the migrants that died in Brooks County prior to 2013. Elmer’s story exemplifies the invisibility of migrants and the trauma endured by families that have no power within the local and state agencies that have failed to follow the state laws.

## Exhumations, processing, and skeletal analyses

In 2013, volunteer exhumations took place at Sacred Heart directed by Baylor University (Dr Lori Baker) and University of Indianapolis (UINDY) (Dr Krista Latham). The exhumations began in areas that contained grave markers indicating “unidentified” and “unknown” remains. A total of 68 remains were exhumed. In total, 45 remains were brought to Texas State University for forensic anthropological analysis and identification efforts, under an effort named *Operation Identification* or OpID. The goal of OpID is to locate, identify, and repatriate unidentified human remains found on or near the South Texas border through community outreach, forensic anthropological analysis, and collaboration with governmental and non-governmental organizations while training the next generation of forensic anthropologists.

**Figure 1.**
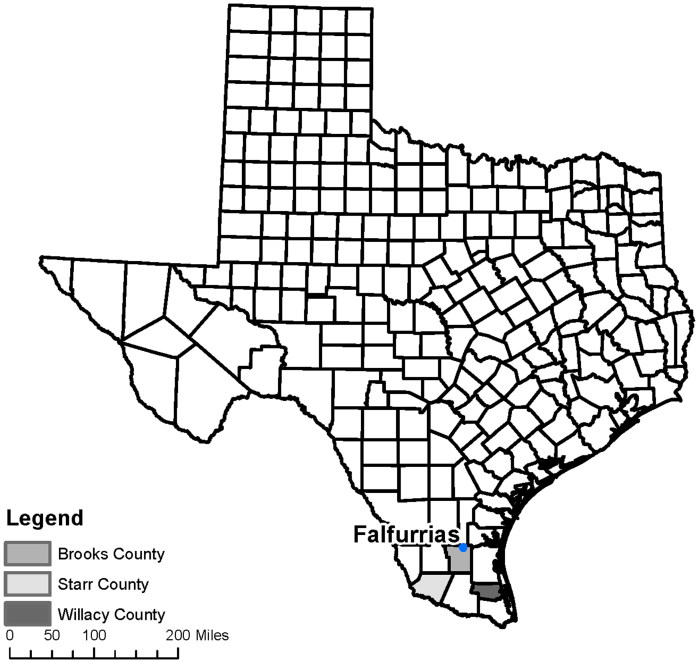
County map of Texas showing locations of Brooks, Starr, and Willacy Counties.

Since 2016, OpID and UINDY volunteers have exhumed remains in Brooks, Starr, and Willacy Counties (Figure 1). In January 2017, OpID began to conduct exhumations in South Texas based on information obtained from the Forensic Border Coalition (FBC) Cemetery Survey Project (https://forensicbordercoalition.org/). Temporary markers are often displaced or have paper documentation which easily fades, and records are not readily available to pinpoint the location of burials of unidentified human remains. Discovering the specific cemeteries where migrants are buried and locating individual burial locations within each cemetery is a challenging task. The FBC has been searching for burials of suspected migrants since 2015 through community outreach, public records requests, and cemetery surveys. OpID works with the FBC to facilitate the exhumations of burials that are suspected to be unidentified migrants.

From the information gathered by the FBC and knowledge gained from previous exhumations concerning general placement of suspected migrants within each cemetery (along the edges of roads or in areas with no permanent headstones), areas of high-probability or regions of interest are noted. Prior to exhumations, each region of interest within a cemetery is mapped using a survey grade mapping instrument, either a Real-Time Kinematic (RTK) GPS system or a total station, and areas of interest are subjected to Ground Penetrating Radar (GPR) survey. Since many of the migrants are buried in body bags rather than coffins or containers, processed GPR data often do not present strong, clearly interpretable anomalies. In addition, soil conditions at some of the cemeteries of interest are poorly suited for GPR scanning. For example, the soil at the Rio Grande City County Cemetery is classified as a very gravelly loam transitioning to cemented material [7], which introduces considerable signal noise due to poor surface contact and varied signal path. Whereas, the soil at Sacred Heart Burial Park in Falfurrias is a fine sandy loam [7], which allows for rapid scanning and fairly straightforward processing.

Systematic use of GPR and geolocation information of burials found through exhumation efforts provide a robust data set for improving the detection of these types of burials in future exhumations. Once areas of interests are mapped and scanned, archaeological excavation techniques are used to exhume remains. While some cemeteries are straightforward, with burials being organized according to rough plot lines at consistent depths, some cemeteries require more extensive archaeological experience to readily observe, interpret, and follow soil disturbances to locate and exhume the unidentified buried there.

Once remains are located, exposed, photographed, and mapped using GPS they are removed from the ground and placed inside a new body bag and given a unique case number. Next, the remains are subject to a field intake which includes preliminary investigation of the contents of the body bag or other burial container. During intake, an external examination is completed which includes documentation of the condition of remains, scars, tattoos, evidence of DNA sampling, and personal effects inventory. Documentation includes written notes and photographs.

The outer most body bag is then sealed and placed in a secure location for the duration of field work. Depending on the circumstances in the field and the scale of the exhumations being undertaken, personal effects may be removed from the remains during intake, then bagged and labelled immediately with the case number. This step may be done to expedite processing upon return to the lab. All personal effects are stored in a freezer prior to cleaning in an effort to mitigate any damage which may be caused by decompositional fluid.

All remains are then transported to the Forensic Anthropology Center at Texas State (FACTS). The majority of remains are stored at the Forensic Anthropology Research Facility (FARF) until room is available for processing the remains in the Osteological Research and Processing Laboratory (ORPL). Decompositional research at FARF is conducted only on remains of individuals who have donated themselves or the next of kin has donated and proper consent has been given. FARF acts only as a secure holding facility for exhumed migrant remains. Once the remains are brought to ORPL, a lab intake is conducted prior to processing. This intake consists of opening the body bag in the processing lab, locating and inventorying all remains, removing and photographing any personal effects still with the remains, screening decomposition fluid and/or dirt for small or fragmentary bones or personal effects, and noting the condition of remains. Contemporaneous notes are taken and the entire process is photo-documented. Any personal effects discovered during lab intake procedures are bagged, labelled, and placed in the freezer with all other unprocessed personal effects to prevent further deterioration.

The bones are placed in a steam-jacketed kettle with water and a mild detergent to help loosen remaining soft tissue. A small amount of disinfectant is also added to the water to kill any harmful microorganisms which may be present. Upon removal from the kettle, the bones are cleaned at an autopsy sink and laid to dry for skeletal analysis. After skeletal analysis, a left fifth metatarsal is removed for DNA profiling. If a metatarsal is not available, then a vertebra is selected. If the case consists solely of a cranium, a section of the petrous portion is cut for DNA sample submission. If no other small bones are available, a section of a long bone is submitted. Metatarsals have thus far have been successful at yielding full STR profiles with only a few cases requiring resubmission. Once the remains have been analyzed and a DNA sample obtained, they are placed in a cardboard box and curated pending identification. The personal effects are washed in five-gallon buckets with plungers and line dried. Once dried, the clothes and personal effects are photographed and uploaded to the National Missing and Unidentified Persons System (NamUs, www.namus.gov) along with any information gleaned through skeletal analysis.

The skeletal analyses and subsequent reports follow best practices outlined by the Scientific Working Group in Forensic Anthropology as the Organization of Scientific Area Committees for Forensic Science guidelines are not yet available. Further, craniometric data are collected using 3Skull [8] and a Microscribe digitizer, along with Zobeck postcranial measurements and pelvic trait expression [9]. ADBOU [10] is used to provide an age estimate based on transition analysis and all trait scores are archived [10]. When possible, the revised version of Fully is used to calculate stature [11], if the skeleton is less complete stature is estimated using FORDISC [12]. The completion date of each workflow task is documented in a Microsoft Access database.

Unlike burials from other counties, many of the individuals exhumed from Brooks County have been re-associated with recovery reports from the sheriff's office, providing the location and date of where the remains were found prior to burial (e.g. a specific ranch).

Circumstances regarding the case, including the location the remains were found, is included in the report and uploaded to NamUs. Many families know the date last known alive and may know an approximate last-known-alive location because of cell phone conversations or from geolocation information from photos sent to family members. This information is important to archive if a genetic association is made, as all antemortem and postmortem information along with the DNA report must be reviewed for consistency. Therefore, obtaining case information along with missing persons’ information from the South Texas Human Rights Center and from the local sheriff’s office has been extremely important in facilitating identifications.

Because OpID has standard data collection procedures, data are then available that can help address broad research questions concerning the overall health of these migrants and where they come from in addition to potentially providing supportive evidence for why they migrant (e.g. poverty or violence, or both). For example, graduate student research has addressed limb proportionality and health [13], geographical variation in body mass [14], as well as the utility of isotope analysis and isoscape modelling to predict potential country of origin [15]. Furthermore, the data collected can be used in conjunction with positive identifications to generate and improve the accuracy of current methods used to estimate age, sex, geographic origin, stature, and overall health, which will help future identification efforts.

## International borders and federal policies: issues with genetic associations and positive identifications

Working within the United States, there are federal tools available for missing and unidentified persons that can be utilized both directly and indirectly by forensic anthropologists, specifically NamUs and Combined DNA Index System (CODIS). All cases exhumed from South Texas that are considered part of OpID are input into NamUs. For unidentified persons (UPs), NamUs contains information regarding where and when the remains were found, demographic characteristics, photographs of clothing, and other personal effects (if available), whether DNA has been submitted to CODIS, a dental chart, as well as other information pertaining to the case. For missing persons (MPs), NamUs contains the full suite of information found on a missing person’s report, including where and when the person was last known alive, basic demographics and unique characteristics, a facial photograph, known medical conditions, surgeries and/or implants, a dental chart, and whether a family DNA reference sample (FRS) has been submitted to CODIS.

CODIS is made up of a series of databases that contain DNA from missing individuals or their family, unidentified human remains, and DNA from criminal offenders, arrestees, and crime scenes. CODIS is further broken down into local DNA index system (LDIS), state DNA index system (SDIS), and national DNA index system (NDIS) databases. CODIS was founded and is owned by the FBI and in Texas, state laws require DNA from all unidentified human remains be sent to the University of North Texas Center for Human Identification (UNTCHI) for DNA profiling and upload to CODIS. Texas has one of the few labs in the country accredited to upload unidentified human remains DNA profiles into CODIS. Because UNTCHI receives money from the federal government (National Institute of Justice), they provide this service for free to all states. CODIS software works to find genetic associations by routinely comparing the short tandem repeats (STR) profiles from the unidentified human remains sample to FRS and the criminal offender database. Although, according to CODIS policy, FRS genetic profiles are never compared against criminal offender, arrestee, and crime scene profiles.

In order for a family to submit DNA for inclusion to CODIS, a law enforcement officer from an investigative agency (including medical examiners) must be present at the time the DNA sample is obtained (www.namus.gov). Law enforcement presence may deter members of the undocumented population from reporting a missing person. Additionally, undocumented individuals may also be reluctant to have their DNA in a database owned by the federal government. Furthermore, families in Latin America that are missing a loved one are unable to get their DNA sample into CODIS for continuous cross-referencing under current regulations and protocols. Therefore, while DNA samples are submitted to CODIS from unidentified human remains, CODIS lacks the many FRS necessary to generate genetic associations that will lead to positive identification.

Each year, the Harris County Institute of Forensic Sciences hosts a *Missing in Harris County Day* that is co-sponsored by NamUs. At this event, families can report missing persons and submit a DNA sample that will go into CODIS. At this event, non-governmental organizations (NGOs), the Migrant Rights Collective and the South Texas Human Rights Center, work with Harris County to provide a safe space for anyone to report a missing person known to have gone missing at or around the South Texas border. The NGOs help to spread the word within the community that the *Missing Day* provides a safe space where citizenship status is not questioned. Here the Migrant Rights Collective and South Texas Human Rights Center obtain a missing person’s report from the family and then the family gives consent to have their DNA sample taken and uploaded to CODIS. OpID has a total of 13 identifications resulting from genetic associations within CODIS. Over half of the CODIS genetic associations are direct results of the non-governmental associations work during the *Missing Day*.

The remaining identifications from OpID come from working with another NGO, the Equipo Argentino de Antropología Forense (EAAF). The EAAF has been working along the US/Mexico border since 2009 [16]. They also work in Chiapas, Mexico, Honduras, and El Salvador collecting FRS samples from families of the missing. During an intake, if personal effects suggest a name association (e.g. an identification card is found or a name and phone number written on paper), the EAAF is contacted to inquire if they have a potential FRS on file or if they can get one. Once the FRS is obtained, the EAAF will pay for the genetic profiling and comparison of the FRS and the unidentified human remains sample at a private accredited DNA laboratory.

The EAAF has FRS from families of the missing and CODIS has DNA samples from unidentified human remains. However, due to policy restrictions within the United State federal system, it is currently not possible to conduct, large-scale DNA cross-referencing, despite 5 years of negotiations. If the DNA profiles from both systems were routinely cross-referenced on a large scale, hundreds if not thousands of individuals could be identified and repatriated to their families.

## Collaborative approaches to identifications

In the fall of 2013, student volunteers began to wash clothing. Several days later, a brown plaid shirt and black sneakers associated with case number 0387 were drying in the processing lab. At that time, there were only eight NamUs entries for missing persons last known alive in Brooks County. In looking over the MP reports in NamUs, one entry was for Elmer. Elmer’s NamUs entry provided witness testimony from a coyote (a paid guide to help migrants cross the border and arrive at a specified destination) who stated that Elmer had a hurt leg and someone tied a brown plaid shirt around his knee. Elmer was left underneath a tree near Falfurrias because he could no longer walk and he was dehydrated.

Based on the description of the brown plaid shirt, a further comparison of antemortem (MP) information was checked against the anthropology report and the full listing of personal effects. All circumstantial evidence suggested that OpID case 0387 and Elmer were likely one in the same. Elmer had dental records available, which is rare for the migrant cases. Dr James P. Fancher, certified by the American Board of Forensic Odontology, compared the antemortem and postmortem data and concluded they were one in the same. While a dental identification is sufficient in most cases, the EAAF had a DNA sample on file and chose to pursue a DNA identification. Elmer’s family reported him missing in August of 2012 and the EAAF collected DNA from the family at that time. After discussion with EAAF, a bone sample was sent to a private lab for genetic profiling and compared to Elmer’s FRS. All costs associated with genetic analyses were paid for by the EAAF. Based on the genetic comparison, along with the comparison of antemortem and postmortem data and a forensic odonotology exam, a positive identification was concluded, giving Elmer his name back.

Because the Justice of the Peace (JP) has jurisdictional authority within Brooks County, an identification report was written by the EAAF, and a request was submitted to the JP in charge of the case to approve and sign the identification report so the name could be legally changed on the death certificate and Elmer could be repatriated to his family in El Salvador. The El Salvadoran consulates in the cities of Houston and McAllen both assisted with the logistical issues and the finances associated with repatriation.

The majority of Texas counties are under jurisdiction of the JP as there are only 13 county medical examiner offices in the state. While a medical examiner can provide autopsy and identification services and has the authority to make a positive identification, it can be more difficult for the 241 counties outside of the medical examiner system, especially for the counties in South Texas faced with a high death toll. With regard to migrant deaths in South Texas, successful identification and repatriation require collaboration between multiple agencies, institutions of higher education, local and international non-governmental organizations, local law enforcement, USCBP, and consulates.

## Back to Brooks County: more exhumations

To date, three large-scale exhumations have taken place in the Sacred Heart Burial Park in Brooks County. The first in May of 2013, the second in June of 2014, and the third in January of 2017. The first two exhumation seasons focused on areas where temporary burial markers indicated the location of unidentified burials and ultimately those two field seasons yielded 122 set of human remains; an additional 44 set of human remains were recovered from a funeral home storage area. From 2013 through 2014, OpID was 100% volunteer based and while casework and identifications advanced, the work was slow. In 2015, OpID received funding to hire a post-doc and part-time lab assistant to reduce the backlog of processing and casework; the amount of case reports written tripled with the hires. Funding is not guaranteed each year, but has been steady since 2015. At this time, there are 270 cases at Texas State associated with OpID.

In January of 2017, OpID and UINDY conducted another field season at Sacred Heart Burial Park. A total of eight markers were visible and guided where the work would begin. In one exhumation area, approximately the size of an average plot, with a single marker present, three individuals were recovered. Several days into field work, a groundskeeper who worked for the county informed the authors that there were several other areas that contained burials and indicated their location. One such area was situated at the intersection of two cemetery roads and temporary burial markers were no longer present. The groundskeeper indicated he knew three people were buried there, roughly in a row, because his riding lawnmower pulled the markers out of place and he forgot to replace them. Geophysical data were collected using GPR. Once excavated, this area which reportedly contained the remains of three individuals, actually contained eight.

**Figure 2.**
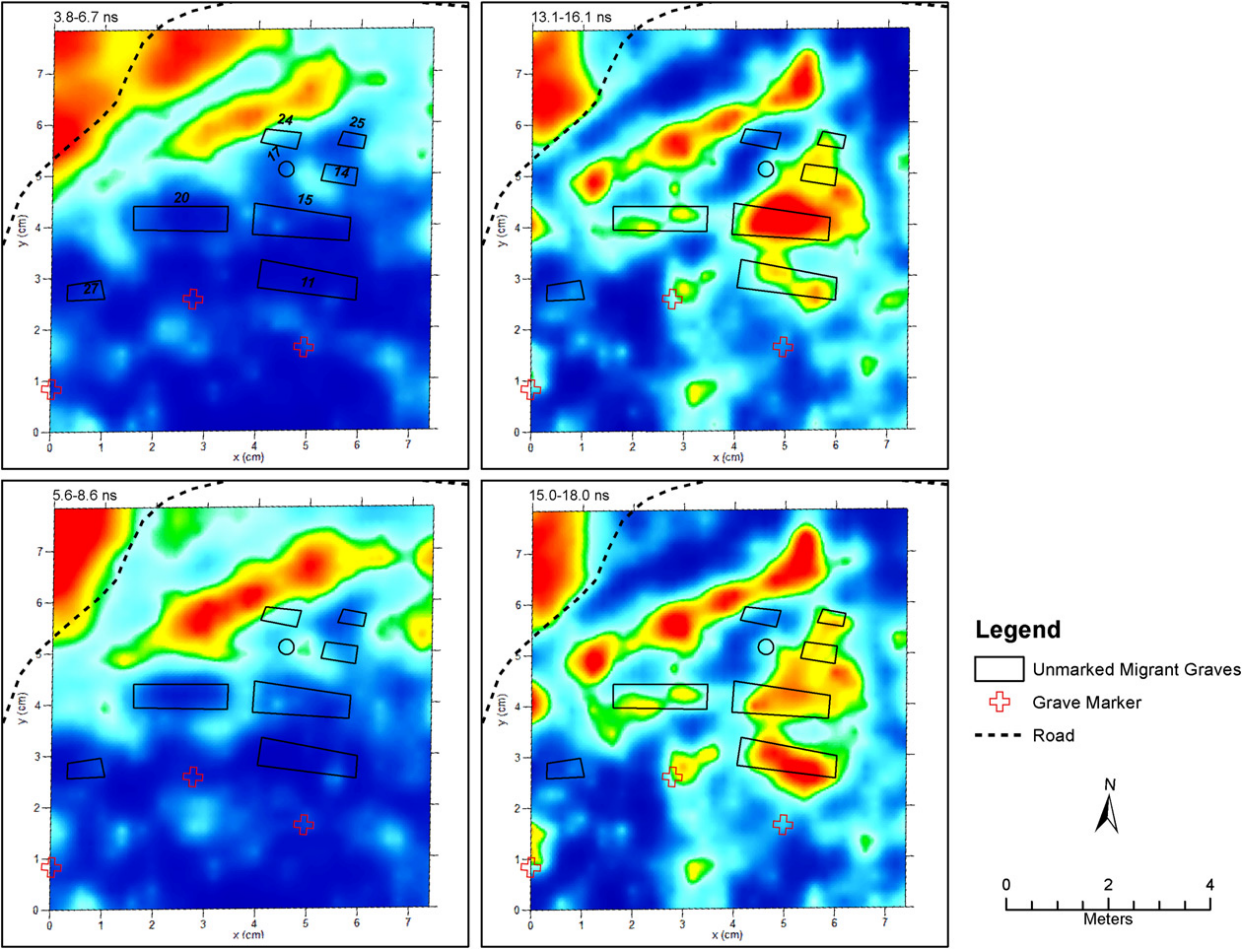
Processed Ground Penetrating Radar (GPR) data from Area 2 South in Sacred Heart Burial Park. The data were processed in GPR- Slice based on 25 cm transects using a 400 mHz Geophysical Survey Systems. Inc. (GSSI) antenna. The two left images represent near surface anomalies and the two images to the right represent anomalies at depth. The black lines represent the unmarked burials.

An example of the combination of GPR data with high-resolution GPS mapping of this area is presented in Figure 2. The GPR data have been minimally processed to define subsurface anomalies and known surface features. To highlight the intricacies of the GPR patterns evident in the Sacred Heart Burial Park four time slices are provided of the processed data, which correspond to four depth ranges. The first two images on the left side of the figure represent the near surface patterns and the two images on the right side of the figure represent time slices at the depth of the burials. The burial locations are outlined on all images and labelled in the upper left image. The road surface presents a consistent stacked anomaly in the upper left corner of all images and there appears to be a waterline that runs parallel to the road boundary. A pattern observed in the upper time slices of the Sacred Heart GPR data is what appears to be burial pit boundaries for several interments (#11, 13, 15, 25 and 27). These present as voids or “quiet areas” in the GPR data. In the deeper time slices, anomalies presented by the burials are not consistent. For example, burials 11 and 13 produce strong signals but clearly show that the burials occur at different depths. These burials were found in body bags in a particleboard box [13] or on a particle board base [11]. Burial 15 presents a bilobate anomaly and these remains were recovered in a body bag with a board underneath as well. The remaining burials, which lack any response or present small anomalies, varied in completeness and burial container.

The other area pointed out by the groundskeeper still had three temporary markers in place. These markers were recorded using GPS to be exhumed during a later field season. At the end of the January field season, 24 individuals were exhumed, and additional areas of interest were located, while initially thinking only eight burials would be exhumed. Towards the end of the field season, a man approached MKS and told her he dug many of the graves of the unidentified by hand and indicated other areas of interest that need to be exhumed. Based on this informant testimony, what was learned during the 2017 field season (location of burials along edges of roads), and the fact that there were at least 6 additional years of burials within Sacred Heart prior to 2012, more extensive/exhaustive search strategies were needed.

**Figure 3.**
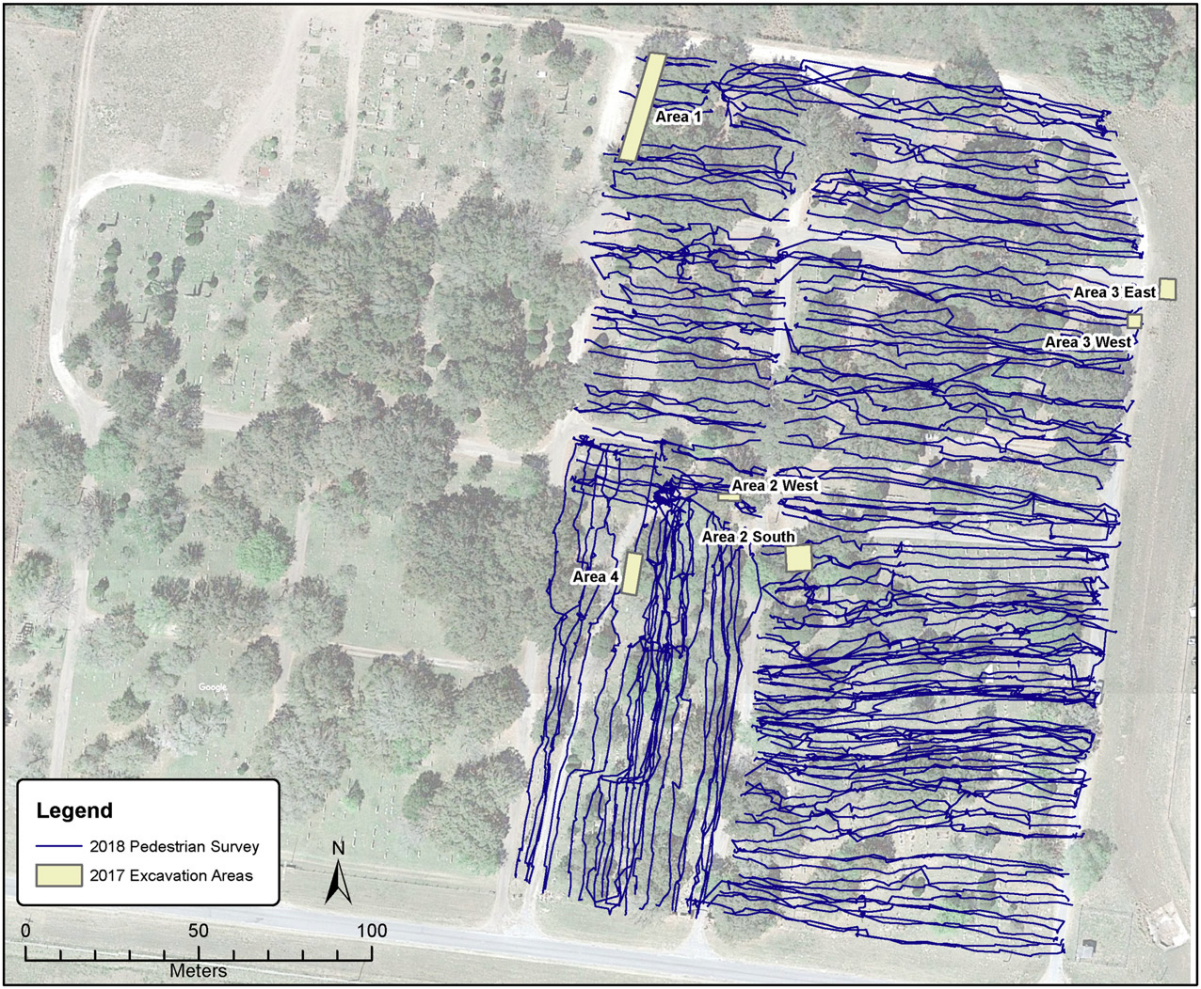
A composite map of 2017 excavation areas and the combined GPS tracklogs for the 2018 pedestrian survey of Sacred Heart Burial Park. The base map is a tiled Google Maps aerial image downloaded on March 10 2018.

In March of 2018, a pedestrian search of the entire cemetery grounds was conducted (Figure 3), during which OpID recorded approximately 40 areas of interest for undocumented burials. The pedestrian survey entailed the use of handheld GPS units with tracklogs activated. Any areas or markers of interest were flagged and recorded using a high-resolution GPS system. The tracklogs from all GPS units were downloaded and plotted to ensure that all areas of Sacred Heart Burial Park were evenly examined. During this survey, high-probability areas were identified and GPR investigations of these areas have been initiated, including one of the largest open areas in the cemetery.

During the pedestrian survey another informant (community member) indicated that he remembered multiple burials of unidentified people in one of the areas already identified by OpID as high probability requiring additional investigation. Due to the preponderance of informant testimony, unaccounted for remains based on Sherriff’s office records, and GPR data currently being analyzed, subsequent exhumations will be planned at Sacred Heart Burial Park to ensure that all unidentified migrants buried there are afforded due effort towards identification, as prescribed under the law.

## Conclusion

The large number of deaths occurring along the US-Mexico border represents a growing humanitarian crisis in both Arizona and Texas. After the attempt to secure the border using the strategy of "Prevention through Deterrence" initiated in 1994, migrants trying to enter the United States were pushed out of urban areas and funnelled into remote and dangerous terrain, resulting in mass fatalities in the region. For Texas, the epicentre of this crisis is situated in Brooks County, near the location of a US Customs Border Patrol Checkpoint. Due to the relatively small population within the county, death investigation falls to the Justice of the Peace. Although the Texas Code of Criminal Procedures Chapters 49 and 63 requires Medical Examiners and Justices of the Peace to conduct an inquest, submit DNA for identification purposes, and record the final disposition of any unidentified remains, the law was not routinely followed in Texas, particularly regarding suspected migrant remains.

Because of the fragmented system in Texas and failure to follow state laws, many unidentified human remains were not analyzed by forensic specialist (pathologist or anthropologist) and were buried without DNA sampling. In these cases, exhumations are necessary although the question of where to exhume and how many are in need of exhumation is a difficult question to answer. To locate migrant burials, it is necessary to use a multidisciplinary approach, including ethnographic research methods to ascertain memory recall, geophysical and archaeological approaches, and historic and public records searches. Furthermore, a transnational and collaborative approach to facilitate positive identification is necessary as not one institution or agency could solely take care of locating, exhuming, identifying, and repatriating unidentified human remains in Texas. To date, 29 individuals have been positively identified through this multi-disciplinary and collaborative approach.
